# Morel-Lavallee Lesion: A Case of Lower Extremity Internal Degloving Injury

**DOI:** 10.7759/cureus.33994

**Published:** 2023-01-20

**Authors:** Muhammad Durrani, Jerry Milas, Jonathan Osmolinski, Samaresh Dasgupta, Curt Cackovic

**Affiliations:** 1 Emergency Medicine, Inspira Medical Center, Vineland, USA

**Keywords:** teaching in emergency medicine, radiology medical education, point-of-care-ultrasound, morel-lavallee lesion, emergency medicine and trauma

## Abstract

A 22-year-old female patient was seen in the emergency department with a two-week onset of progressively worsening pain and swelling to the medial aspect of her distal left femur. The patient was involved in an automobile versus pedestrian accident two months prior sustaining superficial swelling, tenderness, and bruising to the affected area. Radiographs revealed soft tissue swelling without osseous abnormalities. Examination of the distal femur region revealed a large, tender, ovoid area of fluctuance with a dark crusted lesion and surrounding erythema. Bedside ultrasonography revealed a large anechoic fluid collection in the deep subcutaneous plane with mobile internal echogenic debris which was suspicious for a Morel-Lavallee lesion. The patient underwent contrast enhanced CT of the affected lower extremity demonstrating a 8.7 cm x 4.1 cm x 11.1 cm fluid collection superficial to the deep fascia of the distal posteromedial left femur, confirming the diagnosis of a Morel-Lavallee lesion. A Morel-Lavallee lesion is a rare, post-traumatic degloving injury that results in the separation of the skin and subcutaneous tissues from the underlying fascial plane. The resultant disruption of the lymphatic vessels and underlying vasculature leads to progressively worsening hemolymph accumulation. If not recognized and treated in the acute or subacute period, complications can ensue. Complications of Morel-Lavallee include recurrence, infection, skin necrosis, neurovascular injury, as well as chronic pain. Treatment is based on the size of the lesion and ranges from conservative management and surveillance for small lesions to percutaneous drainage as well as debridement, sclerosing agents, and surgical fascial fenestration approaches for larger lesions. Additionally, the utilization of point-of-care ultrasonography can help in the early identification of this disease process. This is important as a delay in diagnosis and subsequent treatment of this disease state is associated with long-term complications.

## Introduction

A Morel-Lavallee lesion is a "closed, traumatic, soft-tissue degloving injury commonly associated with high-energy trauma" [1]. The injury results in the "separation of the skin and subcutaneous fat from the underlying fascial plane" [2]. This rare disease process is most commonly encountered at the greater trochanter, pelvis, and femur, though other anatomic sites may be affected [3]. The true incidence of Morel-Lavallee lesions is not yet established. Case series have estimated an incidence rate of 4% in specific populations, yet these are likely to be underestimated [4]. Studies have noted a male-to-female predominance, which is likely due to the male predominance seen in polytrauma [5]. Morel-Lavallee lesions develop due to traumatic shearing "of the skin and subcutaneous fat from the underlying fascial planes, superficial to the underlying musculature" [2]. This results in the disruption of the transaponeurotic vasculature and lymphatic channels in that anatomic location [1, 5]. The disruption of the capillaries and lymphatic channels allows lymph and blood to accumulate and form a progressively worsening hemolymphatic cavity over time. Morel-Lavallee lesions can be "identified within hours to days after the inciting trauma, but up to one-third of patients present months or years after the initial injury" [3]. Typically, examination reveals local tenderness on palpation, fluctuance, as well as cutaneous hypermobility of the injured skin [3, 6]. Additionally, decreased skin sensation is also typically noted over the area of injured skin. This is secondary to a disruption of cutaneous nerves as a result of the shearing injury in Morel-Lavallee lesions [3, 6]. If not recognized and treated early; a Morel-Lavallee lesion will progress to an inflammatory state due to hemosiderin and granulation tissue deposition with formation of a fibrous capsule. This progressively growing fibrous capsule may lead to complications such as recurrence, secondary infection, skin necrosis, neurovascular compromise, as well as chronic pain [3]. A Morel-Lavallee lesion is primarily a clinical diagnosis, but imaging modalities help to further characterize the lesion and to rule out other competing diagnoses [7]. Although CT may be used in the initial evaluation of these lesions due to its ease and availability in the emergent setting, MRI is considered the imaging modality of choice [3]. Treatment is based on the chronicity and size of the lesion. Treatment ranges from conservative management to aspiration, incision and drainage, debridement, as well as surgical approaches, with or without the use of sclerosing agents [3]. Early identification and treatment are essential in order to avoid the significant morbidity associated with delayed diagnosis. 

## Case presentation

**Figure 1:**
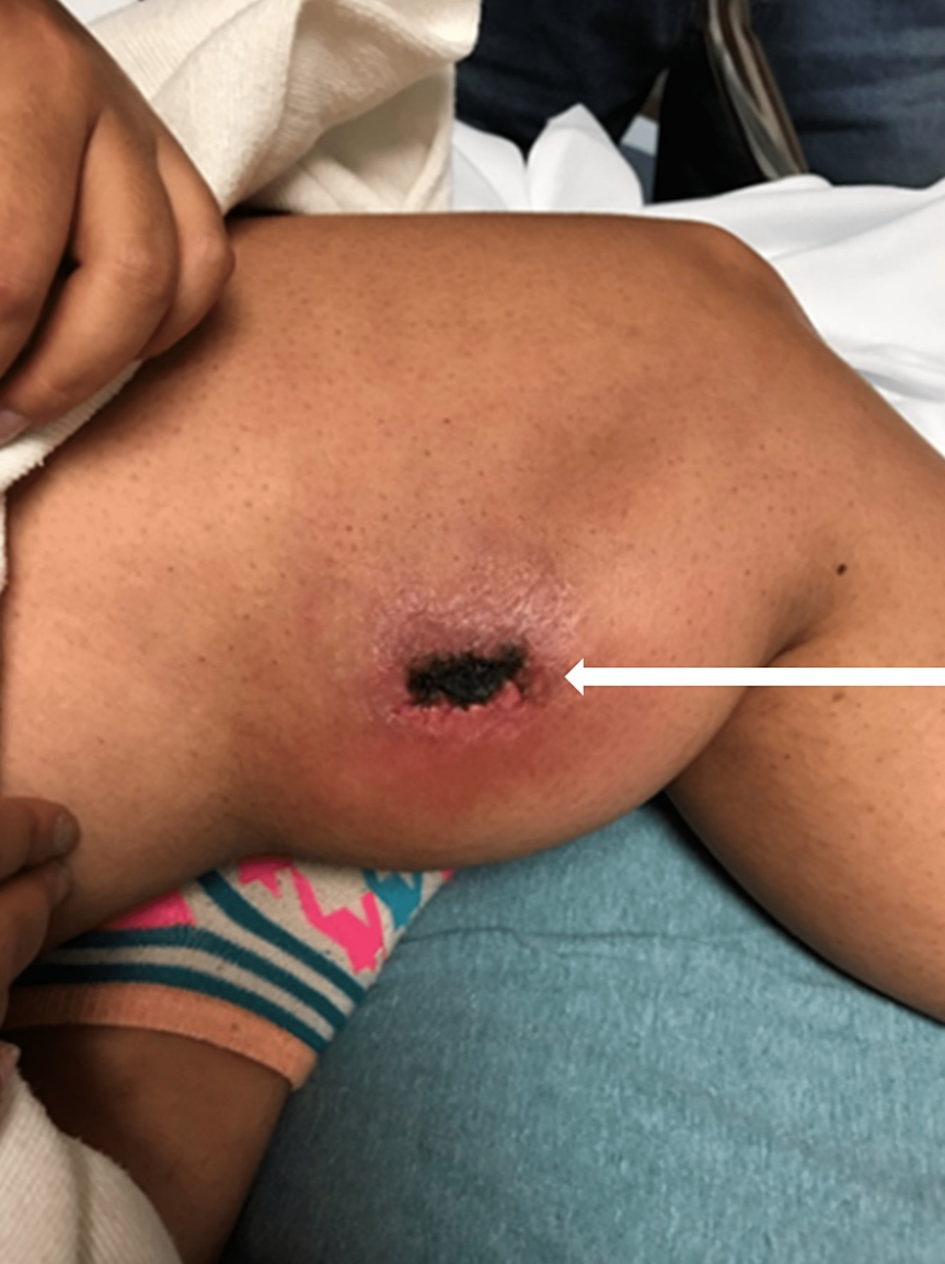
Large, tender, ovoid area of fluctuance and erythema with a central necrotic lesion (white arrow).

A 22-year-old female patient without a significant past medical history presented to the emergency department with a two-week onset of progressively worsening pain and swelling to the medial aspect of her distal left femur. The patient was involved in an automobile versus pedestrian accident two months prior in which the patient’s left lower extremity was pinned between a freeway guardrail and the front bumper of a vehicle traveling at moderate speed. The patient was seen in the emergency department at the time of injury and complained of a tender area of swelling and bruising to the medial aspect of their left femur region. Radiographs were obtained, which showed soft tissue swelling without acute fractures or other abnormalities. The patient was discharged with a diagnosis of soft tissue contusion to their distal left femur. The patient noted that over the ensuing two weeks, the medial aspect of her distal left femur region had progressively become more swollen and painful. Additionally, the patient noted the presence of an enlarging central area of skin change described as a “scab” over the affected region. The patient denied any new trauma, recent surgical interventions, recreational drug use, recent medication use, insect, or tick bites. The patient presented with normal vital signs. Examination of the skin over the distal femur region revealed a large, tender, ovoid area of fluctuance with an overlying crusted lesion and surrounding erythema (Figure 1).

**Figure 2:**
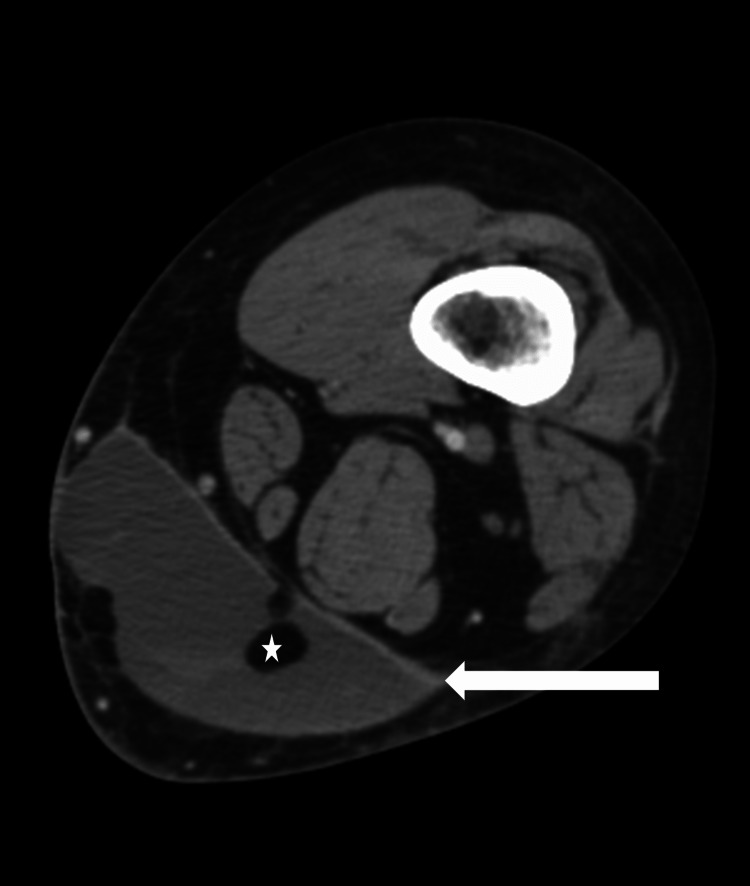
Lenticular shaped 8.7 cm x 4.1 cm x 11.1 cm fluid collection superficial to the fascia of the distal posteromedial left thigh (white arrow) demonstrating locules of fat density material suggestive of lymphatic fluid (white star).

The skin over the affected area was noted to be freely mobile and the patient noted decreased sensitivity to light touch on palpation of the affected cutaneous area. Point-of-care ultrasonography was significant for a large avascular anechoic fluid collection in the deep subcutaneous plane with mobile echogenic debris suspicious for a Morel-Lavallee lesion. The patient underwent a CT scan of the affected lower extremity with contrast to further characterize the lesion. A lenticular shaped 8.7 cm x 4.1 cm x 11.1 cm fluid collection was present superficial to the fascia of the posteromedial left thigh with surrounding inflammatory changes (Figure 2).

The fluid collection was noted to consist of varying ages of blood products and multiple locules of fat density material suggestive of lymphatic fluid. This constellation of findings was diagnostic of a Morel-Lavallee lesion. The patient was admitted to the hospital and required an open incision and drainage in the operating room with placement of a surgical drain. She was discharged with instruction on drain care and close follow-up with surgery. Unfortunately, over the next four to six weeks, she noted multiple recurrences of her lesion requiring repeated surgical drainage.

## Discussion

A Morel-Lavallee lesion is a rare disease state with a high misdiagnosis rate as well as significant morbidity. It is essential to diagnose and treat this clinical condition early since delayed diagnosis and treatment are associated with recurrences of the lesion, secondary infection, skin necrosis, neurovascular compromise, as well as chronic pain. Our patient’s initial presentation of localized skin swelling, tenderness, and bruising after a high-energy blunt traumatic mechanism in a characteristic location may have been a missed opportunity to diagnose a Morel-Lavallee lesion. The delayed diagnosis may have led to the development of a chronic inflammatory type of Morel-Lavallee lesion that is associated with higher rates of complications. We advocate for the early use of point-of-care ultrasonography as an adjunct to the physical examination in the diagnostic work-up of these patients. Incorporation of this modality may allow for a rapid method to enhance the physical examination and potentially strengthen the differential diagnosis of Morel-Lavallee lesions. As the initial symptoms of Morel-Lavallee lesions may overlap with a multitude of clinical conditions, bedside ultrasonography can aid potential diagnostic uncertainty. Ultrasonographic findings of Morel-Lavallee lesions consist of "hypoechoic to anechoic collections located deep to the hypodermis and superficial to the muscular plane" with internal debris that lacks vascularity [1]. Despite Morel-Lavallee lesions being rare, "clinical awareness of this disease entity is crucial for the emergency physician" [8]. "We hope this case report adds to the body of literature on "Morel-Lavallee lesions" and reinforces the importance of point-of-care ultrasonography in "the diagnostic workup of this disease entity” [8].

## Conclusions

Our patient presented with clinical features indicative of a Morel-Lavallee lesion. Yet, due to diagnostic uncertainty, point-of-care ultrasonography was utilized early in the diagnostic workup of our patient. The use of this modality allows us to refine our differential diagnosis and subsequently confirm the diagnosis with advanced imaging. Morel-Lavallee lesions can be an elusive diagnosis with significant diagnostic uncertainty due to their non-specific presentation. Unfortunately, they are associated with significant morbidity when undiagnosed or untreated. For this reason, we feel that clinicians should be aware and vigilant of this disease process. 
